# Association between the *SLC6A11* rs2304725 and *GABRG2* rs211037 polymorphisms and drug-resistant epilepsy: a meta-analysis

**DOI:** 10.3389/fphys.2023.1191927

**Published:** 2023-05-19

**Authors:** Xuemei Hu, Mingyang Zhao, Xue Yang, Dongsen Wang, Qingjian Wu

**Affiliations:** ^1^ Clinical Medical College of Jining Medical University, Jining, Shandong, China; ^2^ Department of Emergency, Jining No. 1 People’s Hospital, Jining, Shandong, China

**Keywords:** drug-resistant epilepsy, single-nucleotide polymorphism, *SLC6A11*, *GABRG2*, meta-analysis

## Abstract

**Background:** Previous studies have shown that *SLC6A11* and *GABRG2* are linked to drug-resistant epilepsy (DRE), although there have been conflicting results in the literature. In this study, we systematically assessed the relationship between DRE and these two genes.

**Methods:** We systematically searched the PubMed, Embase, Cochrane Library, Web of Science, Google Scholar, Wanfang Data, CNKI, and VIP databases. To clarify whether heterogeneity existed between studies, tools such as the Q-test and *I* ^*2*^ statistic were selected. According to study heterogeneity, we chose fixed- or random-effects models for analysis. We then used the chi-squared ratio to evaluate any bias of the experimental data.

**Results:** In total, 11 trials and 3,813 patients were selected. To investigate the relationship with DRE, we performed model tests on the two genes separately. The results showed that *SLC6A11* rs2304725 had no significant correlation with DRE risk in the allele, dominant, recessive, and additive models in a pooled population. However, for the over-dominant model, DRE was correlated with rs2304725 (OR = 1.08, 95% CI: 0.92–1.27, *p* = 0.33) in a pooled population. Similarly, rs211037 was weakly significantly correlated with DRE for the dominant, recessive, over-dominant, and additive models in a pooled population. The subgroup analysis results showed that rs211037 expressed a genetic risk of DRE in allele (OR = 1.01, 95% CI: 0.76–1.35, *p* = 0.94), dominant (OR = 1.08, 95% CI: 0.77–1.50, *p* = 0.65), and additive models (OR = 1.14, 95% CI: 0.62–2.09, *p* = 0.67) in an Asian population.

**Conclusion:** In this meta-analysis, our results showed that *SLC6A11* rs2304725 and *GABRG2* rs211037 are not significantly correlated with DRE. However, in the over-dominant model, rs2304725 was significantly correlated with DRE. Likewise, rs211037 conveyed a genetic risk for DRE in an Asian population in the allele, dominant, and additive models.

## 1 Introduction

Epilepsy is a chronic neurological disease that is very harmful to human health, with a global prevalence of 1% (Zhang et al., 2021) and affecting more than 70 million people worldwide (Thijs et al., 2019). Although multiple antiepileptic drugs (AEDs) may be used alone or in combination, approximately one-third of people with epilepsy are unable to fully control their epilepsy, a phenomenon termed drug resistance (Löscher et al., 2020). Although the pathogenesis of drug-resistant epilepsy (DRE) is not clear, many drug-resistant epilepsy hypotheses have attempted to explain its occurrence (Löscher et al., 2020). Of course, there are some conjectures or hypotheses that some clinical factors are associated with drug resistance (Kalilani et al., 2018). In short, the factors affecting the occurrence of DRE can be roughly divided into environmental and genetic causes. Some environmental factors may be able to be controlled, but many may remain elusive. However, the identification of genetic factors may prove easier, especially with the rapid increase in our knowledge of human genome variation (Sisodiya, 2005).

We consulted DrugBank5.0 (Wishart et al., 2018) and the 2020 Therapeutic Target Database (Wang et al., 2020) and found 115 approved resistant epilepsy dysentery drug targets. One of the targets that affect AEDs is the neurotransmitter systems, and the neurotransmitters include γ-aminobutyric acid (GABA) and glutamate, as they act on the γ-aminobutyric acid type A receptor (GABA_A_R) to maintain brain excitation homeostasis, which plays a key role in inhibiting epilepsy (Macdonald et al., 2004; Maljevic et al., 2019). Because GABA_A_R is widely distributed in the central nervous system and they have the potential for postsynaptic inhibition, the GABA receptor is considered to be a hotspot for idiopathic generalized epilepsy susceptibility and is regulated by therapeutically important antiepileptic drugs (Yu Sun et al., 2021). GABA_A_ receptors consist of four subunits, among which *GABRA1*, *GABRB2*, and *GABRG2* genes encode the most common subunits, α1, β2, and γ2, respectively (Mulligan et al., 2012). Failure of the genes that encode these subunits can affect their expression, leading to epilepsy (Saleem et al., 2022). The GABA_A_ receptor is the main target of antiepileptic drugs, and changes in the GABA_A_ receptor subunit may play a role in antiseizure medication resistance (Bethmann et al., 2008). Therefore, the GABA receptor is selected as a novel method to discuss the relationship between this gene and DRE. In 2011, Kim et al. found a meaningful association between *SLC6A11* and DRE. Similarly, in 2017, Xie et al. found that slc6a11 had no significant correlation with DRE in the Chinese population. Previous animal model studies have also shown that inhibition of GABA transport-3 (GAT-3) increases the concentration of GABA in the environment, leading to reduced neuron firing (Galvan et al., 2005). We can also infer that GAT-3 is a potential target for DRE.

Thus, in this study, we aimed to discuss the possible connections between *SLC6A11*, *GABRG2*, and targeted genetic variation in DRE to provide novel targets and strategies for the treatment of epilepsy in the future.

## 2 Materials and methods

### 2.1 Search strategy

We systematically retrieved data from PubMed, Embase, Cochrane Library, Web of Science, Google Scholar, Wanfang Data, China National Knowledge Infrastructure (CNKI), and China Science and Technology Journal (VIP) databases. The relevant literature was updated on 18 April 2023. Under the guidance of library service experts at Jining Medical University, we formulated a detailed search strategy and implemented the search. The main retrieval strategy of this study was (DRE OR Intractable Epilepsy) AND (SLC6A11 OR rs2304725 OR GAT-3). The complete search strategies for the eight databases are shown in Supplementary Table S1.

### 2.2 Selection criteria

The inclusion criteria were as follows: 1) the type of article included must be a case–control design, 2) the included study investigated two SNPs (rs2304725 and rs211037) in connection with DRE, 3) included studies should provide genotype or allele numbers (Liu et al., 2013), 4) included studies may provide odds ratios (ORs) and 95% confidence intervals (CIs), and 5) the data in the included articles could be calculated to give an OR and 95% CI(Wang et al., 2022). Inclusion criteria for the DRE group (2010 International Anti-epileptic League): patients whose seizures have not been completely controlled with sufficient doses of two or more reasonable DRE. Inclusion criteria for epilepsy in the drug-sensitive group: reasonable use of antiepileptic drugs, according to the longest interval of epileptic seizure in the latest 12 months, three times the longest interval of seizure (≥12 months) without seizures (Fisher et al., 2005). The studies excluded did not meet the inclusion criteria. Based on the aforementioned acceptance criteria, non-conforming documents were excluded.

### 2.3 Data extraction

Two researchers (XH and MZ) extracted the required data separately, and the differences were eliminated through discussion. We extracted information based on the inclusion criteria, including the first author’s name, population, publication year, sample size, the number and frequencies of *SLC6A11* rs2304725 and *GABGR2* rs211037 in the cases and controls, and the OR values and 95% CIs. The detailed information extracted is shown in Table 1 and Table 2 **.**

**TABLE 1:** Main characteristics of rs2304725 and drug-resistant epilepsy in meta-analysis.

Gene	SNP	First author, year	Population	Case	Control	Case genotype			Control genotype			T (case/control)	C (case/control)	OR	95% CI	SE (ln (or))
						TT	TC	CC	TT	TC	CC					
SLC6A11	rs2304725	Xie et al. (2017)	Chinese	192	288	89	73	30	145	105	38	251/395	133/181	0.865	0.657–1.137	0.14
		Kim et al. (2011a)	Korean	200	200	70	97	33	64	98	38	237/226	163/174	1.119	0.845–1.482	0.143
		Long (2014)	Chinese	207	273	82	61	64	97	103	73	225/297	189/249	1.096	0.826–1.456	0.145
		Hidayati (2016)	Chinese	211	211	56	96	59	66	98	47	208/230	214/192	0.811	0.619–1.063	0.138
		Hidayati (2016)	Indian	145	151	25	72	48	32	78	41	122/142	168/160	0.818	0.591–1.132	0.166
		Hidayati (2016)	Malaysian	215	212	67	103	45	60	104	48	237/224	193/200	1.096	0.838–1.435	0.137

Note: SNP, single-nucleotide polymorphism; OR, odds ratio; CI, confidence interval; SE, standard error; (C), Chinese; (I), Indian; (M), Malaysian.

**TABLE 2:** Main characteristics of rs211037 and drug-resistant epilepsy in meta-analysis.

Gene	SNP	First author, year	Population	Case	Control	Case genotype			Control genotype			C (case/control)	T (case/control)	OR	95% CI			SE (ln (or))
						CC	CT	TT	CC	CT	TT							
GABRG2	rs211037	Kumari et al. (2010)	Indian	122	259	66	53	3	137	109	13	185/383	59/135	1.105	0.777–1.573			0.1801
		Balan et al. (2013)	Kerala	240	198	165	66	9	142	47	9	396/331	84/65	0.926	0.649–1.321			0.181
		Kim et al. (2011a)	Korean	200	200	67	97	36	68	97	35	231/233	169/167	0.98	0.740–1.297			0.143
		Butilă et al. (2018)	Romanian	11	49	1	7	3	30	17	2	9/77	13/21	0.189	0.071–0.502			0.499
		Abou El Ella et al. (2018)	Egyptian	54	46	16	26	12	30	16	0	58/76	50/16	0.244	0.126–0.472			0.336
		Gao et al. (2020)	Xinjiang	28	51	9	12	7	17	20	14	30/54	26/48	1.026	0.534–1.971			0.333
		Saleem et al. (2022)	Pakistani	55	88	27	21	7	28	37	23	75/93	35/83	1.912	1.161–3.149			0.011
		Qian (2017)	Guangxi	38	69	5	16	17	21	28	20	26/70	50/68	0.505	0.283–0.902			0.021

Note: SNP, single-nucleotide polymorphism; OR, odds ratio; CI, confidence interval; SE, standard error.

### 2.4 Genetic models

To ensure that interesting findings were not missed because of the different analysis methods used, we also investigated this association under five common genetic models for rs2304725: the allele model (T vs. C), recessive model (TT vs. TC + CC), dominant model (TT + TC vs. CC), over-dominant model (TT + CC vs TC), and additive model (TT vs. CC). Likewise, we also used five common genetic models for rs211037: the allele model (C vs. T), dominant model (CC + CT vs. TT), recessive model (CC vs. CT + TT), over-dominant model (CC + TT vs. CT), and additive model (CC vs. TT).

### 2.5 Statistical analysis

The chi-squared test was used to clarify correlation between DRE and the two SNPs using the R program for analysis (http://www.r-project.org/) (Liu et al., 2013). For the meta-analysis, we determined to use Cochran’s Q test, and the heterogeneity among the datasets was assessed using the following formula: *I* ^*2*^ = (*Q*−*(k*−*1)*)*/Q* × 100%. The *Q* statistic roughly obeyed the *χ* ^2^ distribution, which is the k-1 degrees of freedom (where k is the number of studies) (Liu et al., 2017). When the *p-*value was 50% from Cochran’s Q statistic, the heterogeneity was considered significant in the data (Hu et al., 2017). When *I* ^*2*^ was greater than 50%, and the *p*-value was less than 0.1 (T.J. Higgins JPT et al., 2021), we used the DerSimonian and Laird random-effects model to analyze the data. Conversely, when *I* ^*2*^ < 50%, we used the Mantel–Haenszel or inverse variance fixed-effect model for data analysis. Funnel plots were used to analyze potential publication bias, where an asymmetrical funnel plot indicates the presence of bias, and a symmetrical image represents no bias (Liu et al., 2014).

## 3 Results

### 3.1 Study selection

**FIGURE 1:**
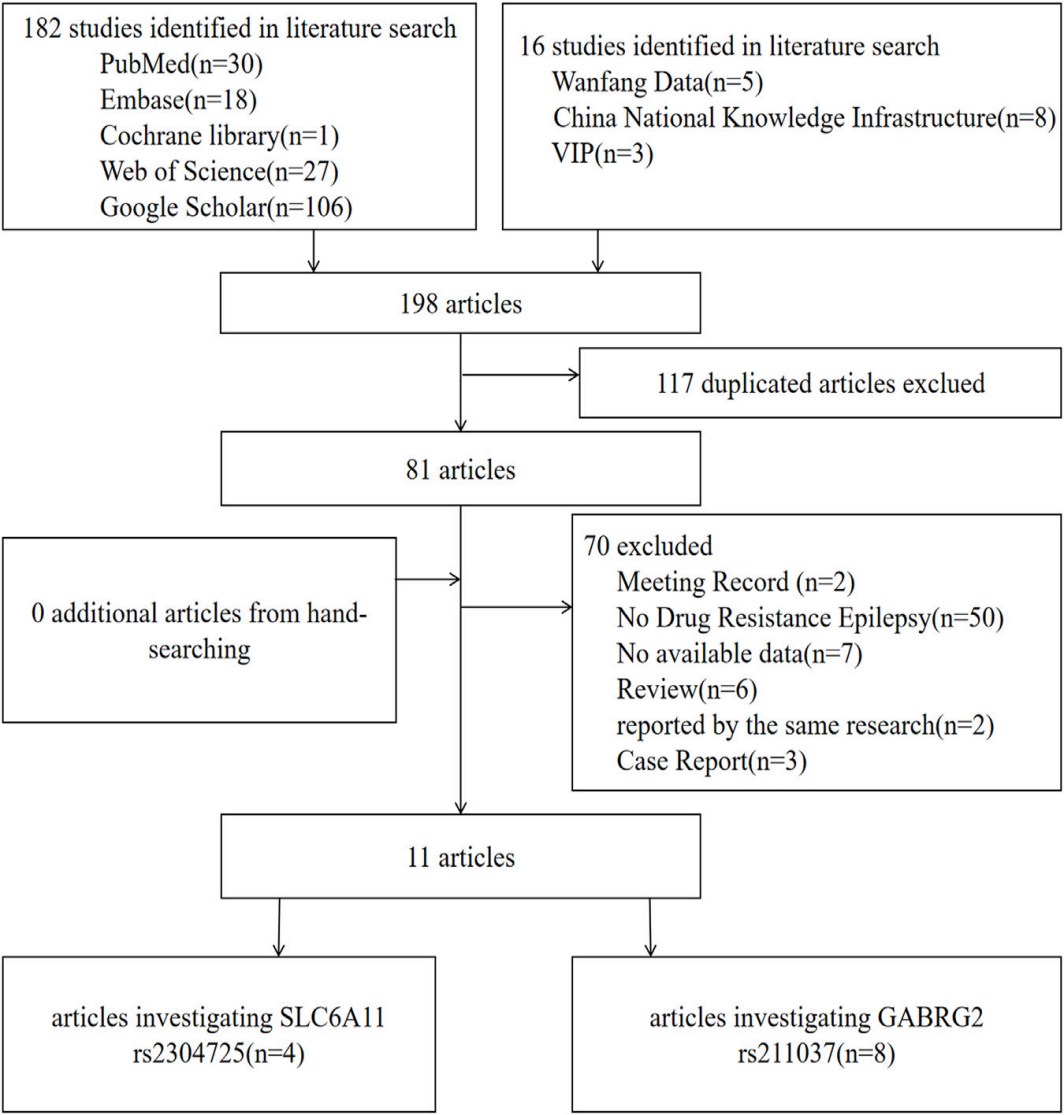
Flow chart of study selection in this meta-analysis.

For *SLC6A11*, according to the search strategy, 40 potentially relevant articles were initially found, and nine articles were excluded for duplication. Furthermore, 27 articles were deleted because they belonged to review articles, case reports, or meeting records, they were reported by the same research group, DRE was absent from the case group, or no data were available. Finally, four articles that suited our study were selected in the meta-analysis (Kim et al., 2011b; Long, 2014; MS, 2016; Xie et al., 2017). Likewise, for *GABGR2* rs211037, eight correlated articles were collected in total (Kumari et al., 2010; Kim et al., 2011b; Balan et al., 2013; Qian, 2017; Abou El Ella et al., 2018; Butilă et al., 2018; Gao et al., 2020; Saleem et al., 2022). One of the articles contained both genes studied in this paper (Kim et al., 2011b). The flow chart of selection of studies in this analysis is shown in Figure 1.

### 3.2 Characteristics of included studies

A total of 2,505 participants were included for *SLC6A11* rs2304725 (the DRE group and the control group contained 1,170 and 1,335 cases, respectively), and 1,708 participants were included for *GABRG2* rs211037 (748 and 960 cases in the DRE and control groups, respectively) in this meta-analysis. The principal characteristics of these studies are shown in Table 1 and Table 2.

### 3.3 Association between the *SLC6A11* rs2304725 polymorphism and DRE

**FIGURE 2:**
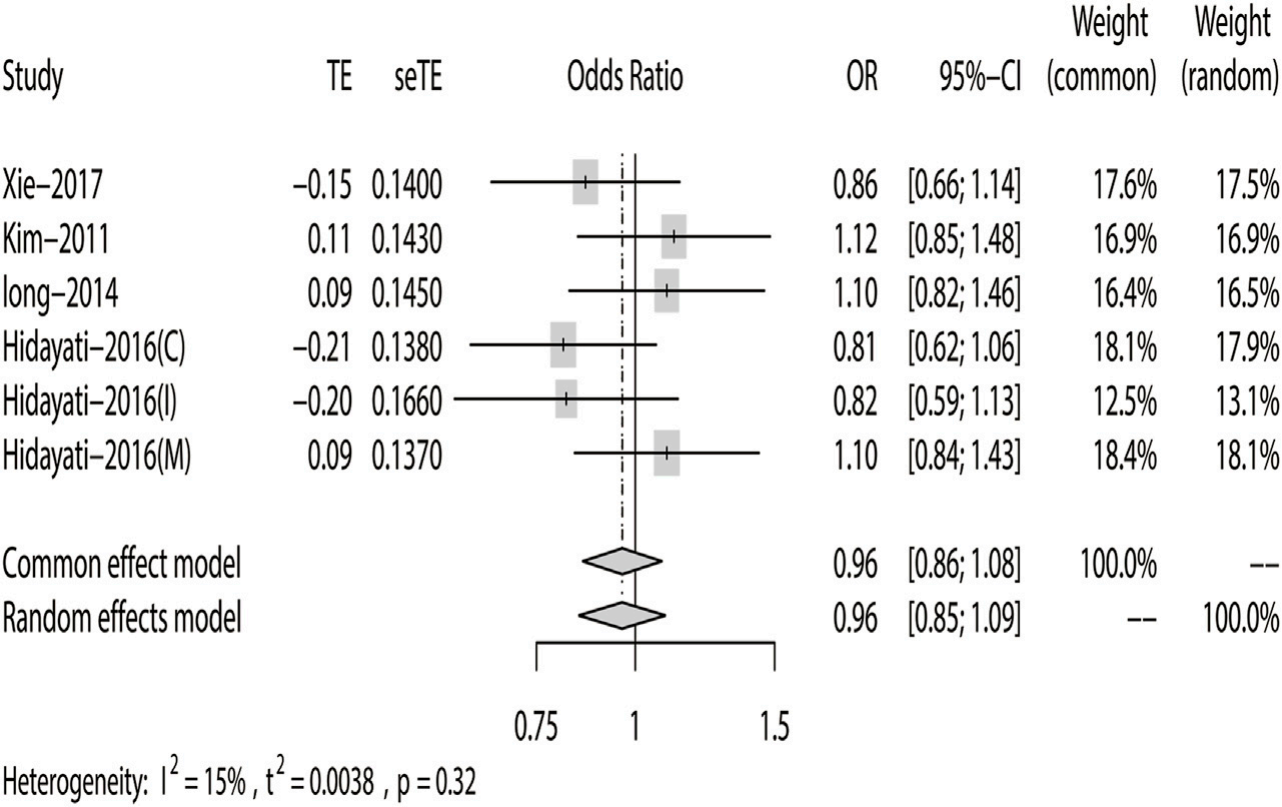
Fixed-effects meta-analysis of the allele model for rs2304725.

**FIGURE 3:**
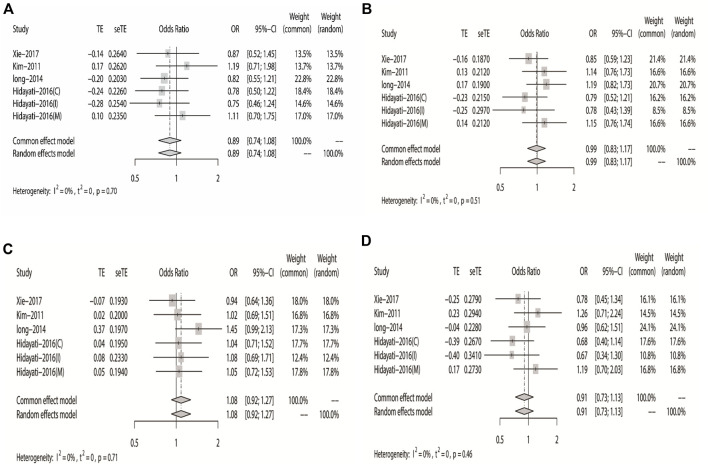
Forest plot of the four models for rs2304725 in this meta-analysis. **(A)** Dominant, **(B)** recessive, **(C)** over-dominant, and **(D)** additive models.

**FIGURE 4:**
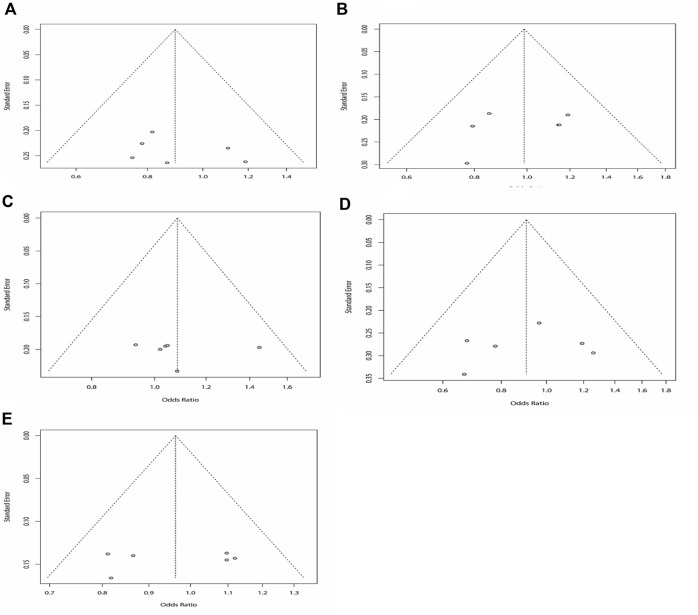
Bias analysis of five models for rs2304725 in this meta-analysis. **(A)** Dominant, **(B)** recessive, **(C)** over-dominant, **(D)** additive, and **(E)** allele models.

According to the results of heterogeneity testing, a fixed-effects model was used to compute the whole OR (*I* ^*2*^ = 15%). The results showed that DRE was unrelated to rs2304725 based on the allele model (OR = 0.96, 95% CI: 0.86–1.08, *p* = 0.52, Figure 2), and the T allele was not correlated with DRE. In addition, we studied the results of the four other models for this gene. Further analyses reported similar results among the four models (additive model: OR = 0.91, 95% CI: 0.73–1.13; recessive model: OR = 0.99, 95% CI: 0.83–1.17; dominant model: OR = 0.89, 95% CI: 0.74–1.08; and over-dominant model: OR = 1.08, 95% CI: 0.92–1.27, Figure 3; Table 3). In conclusion, the results of the over-dominant models showed that *SLC6A11* rs2304725 was significantly correlated with DRE (OR = 1.08), while the other models showed no significant correlation with DRE (OR < 1). Then, to evaluate whether the five genetic models showed potential publication bias, we used a funnel plot and Egger’s test for analysis. The resultant image was a symmetrical inverted funnel, indicating that there was no bias (Figure 4; Table 3).

**TABLE 3:** Analysis of five genetic models’ association of rs2304725 with drug-resistant epilepsy.

	OR	95% CI	*p*-value^*^	I^2^ (%)	Bias	*p*-value^#^
T vs. C	0.96	0.85–1.09	0.32	15	−4.52	0.59
TT + TC vs. CC	0.89	0.74–1.08	0.70	0.0	2.60	0.50
TT vs. TC + CC	0.99	0.83–1.17	0.51	0.0	−2.27	0.47
TT + CC vs. TC	1.08	0.92–1.27	0.71	0.0	0.68	0.91
TT vs. CC	0.91	0.73–1.13	0.46	0.0	−1.65	0.67

Note: OR, odds ratio; CI, confidence interval; *, heterogeneity test; #, publication bias.

### 3.4 Association between the *GABRG2* rs211037 polymorphism and DRE

#### 3.4.1 Meta-analysis of the allele model

**FIGURE 5:**
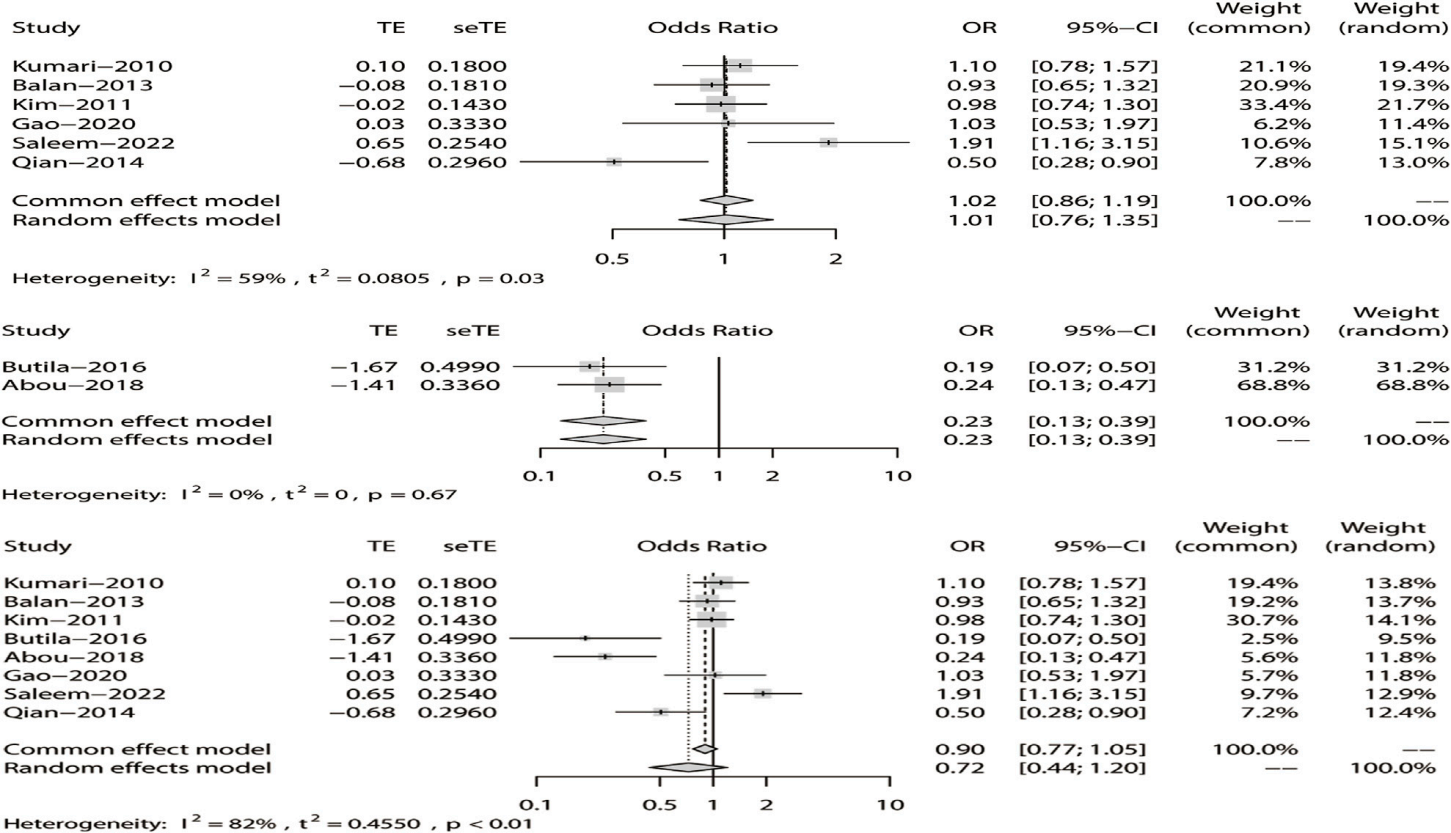
Random-effects meta-analysis of the allele model for rs211037 in the Asian, non-Asian, and pooled populations.

**FIGURE 6:**
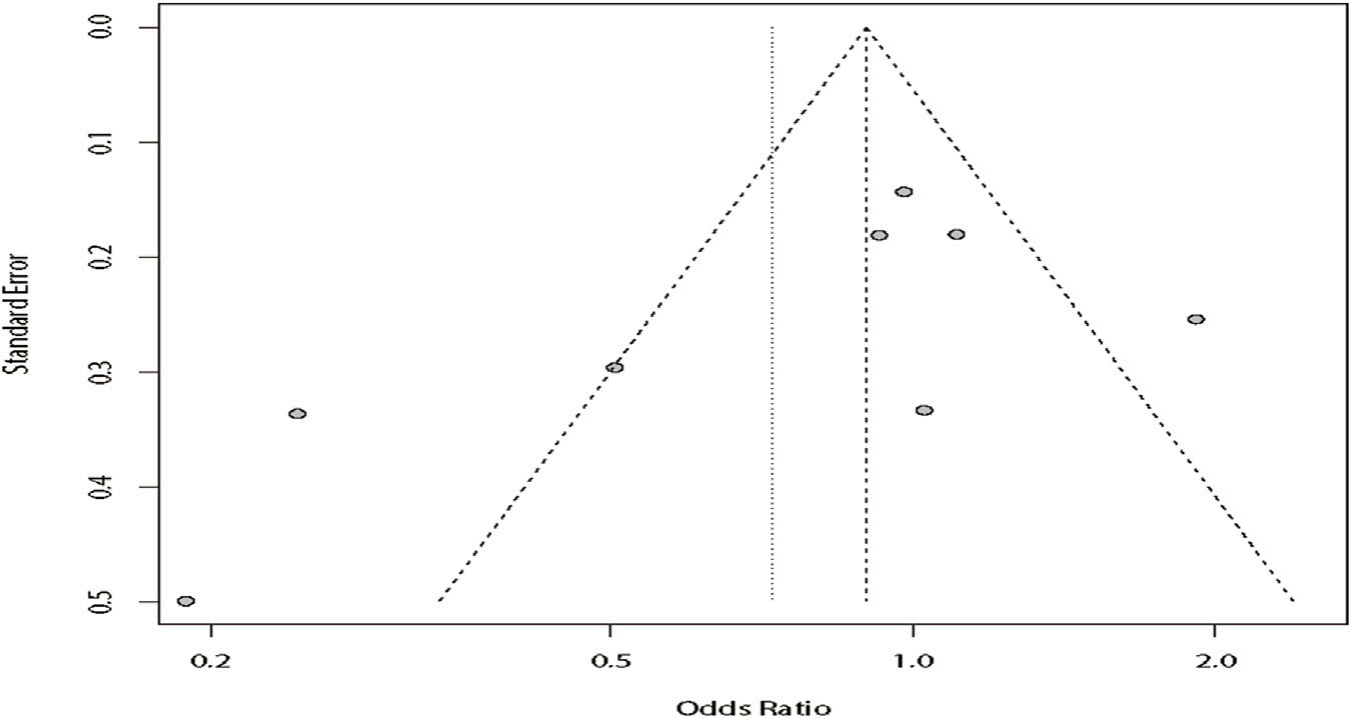
Bias analysis of the allele model for rs211037 in this meta-analysis.

Regarding rs211037, we included eight articles for analysis, and the results indicated that there was heterogeneity in the studies (*I* ^*2*^ = 82%). As there was heterogeneity in the studies (*I* ^*2*^ > 50%), we chose the random-effects model analysis. We conducted subgroup analyses of Asian and non-Asian populations. The allele model test showed that DRE was not related to rs211037 in the Asian (OR = 1.01, 95% CI: 0.76–1.35, *p* = 0.94), non-Asian (OR = 0.23, 95% CI: 0.13–0.39, *p* < 0.01), and pooled populations (OR = 0.72, 95% CI: 0.44–1.20, *p* = 0.21) (Figure 5). Our results suggested that rs211037 was linked to DRE in the Asian population (OR = 1.01) but not linked to DRE in the non-Asian population (OR = 0.23). Next, we tested for publication bias in the subgroup analysis and found that there was no publication bias (Table 4; Figure 6).

**TABLE 4:** Analysis of different models’ association of rs211037 with drug-resistant epilepsy.

Genetic model	Genotype	Asian			Non-Asian			Pooled				
		OR	95% CI	*p*-value^*^	OR	95% CI	*p*-value^*^	OR	95% CI	*p*-value^*^	Bias	*p*-value^#^
Allele	C vs. T	1.01	0.76–1.35	0.03	0.23	0.13–0.39	0.67	0.72	0.44–1.20	<0.01	−3.67	0.127
Recessive	CC vs. CT + TT	0.99	0.80–1.23	0.13	0.19	0.09–0.42	0.28	0.70	0.40–1.22	<0.01	−2.45	0.130
Dominant	CC + CT vs. TT	1.08	0.77–1.50	0.19	0.08	0.02–0.40	0.53	0.90	0.51–1.58	0.01	−1.48	0.302
Over-dominant	CC + TT vs. CT	0.92	0.74–1.14	0.91	0.49	0.24–0.97	0.43	0.87	0.71–1.07	0.19	−1.45	0.070
Additive	CC vs. TT	1.14	0.62–2.09	0.06	0.02	0.00–0.16	1.00	0.64	0.23–1.75	<0.01	−2.37	0.146

Note: vs., versus; OR, odds ratio; CI, confidence interval; *, heterogeneity test; #, publication bias.

#### 3.4.2 Meta-analysis with the recessive model

**FIGURE 7:**
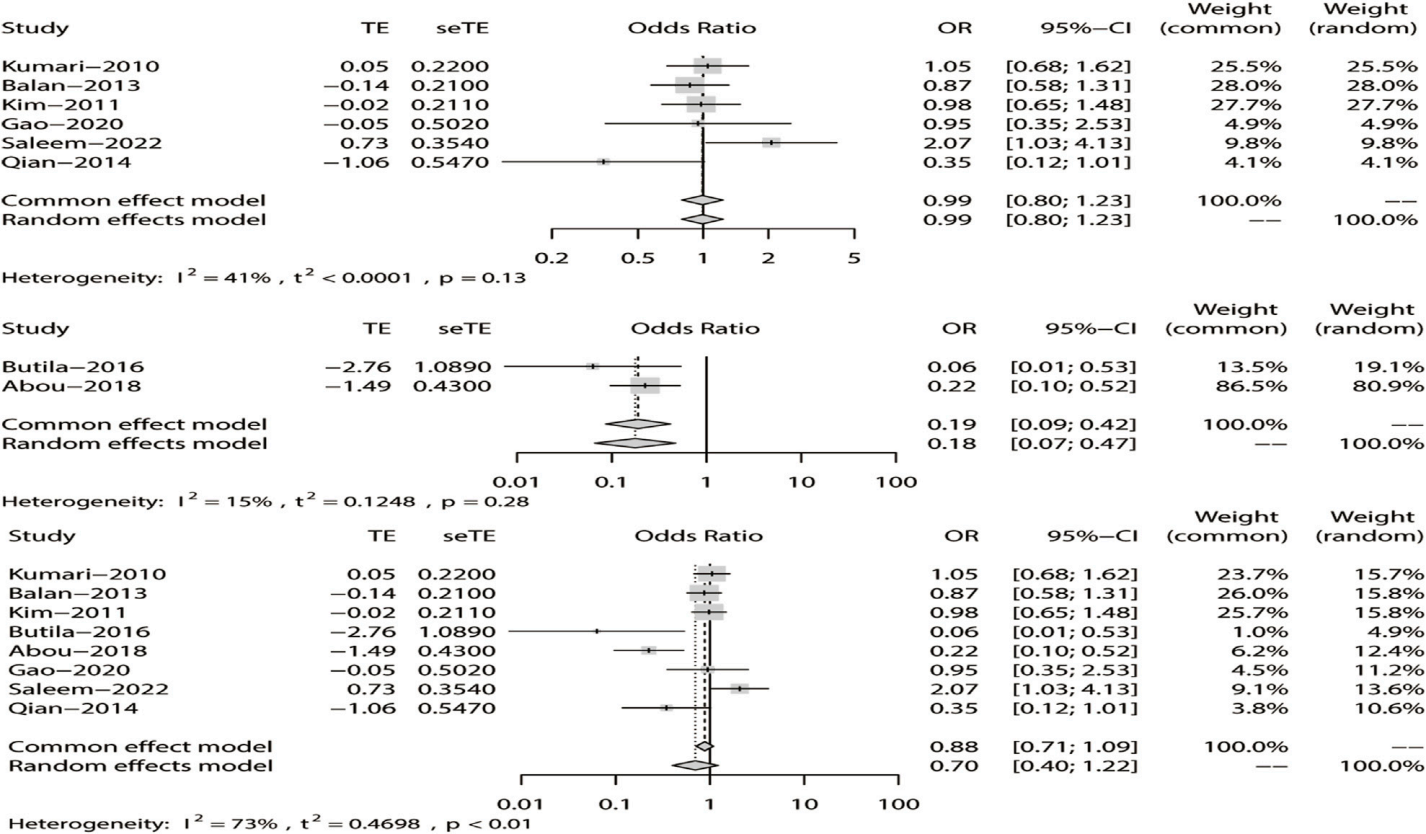
Random-effects meta-analysis of the recessive model for rs211037 in the Asian, non-Asian, and pooled populations.

**FIGURE 8:**
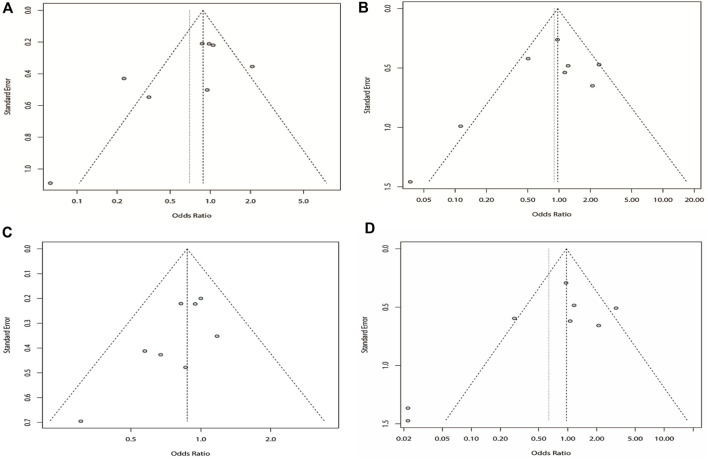
Bias analysis of four models for rs211037 in this meta-analysis. **(A)** Recessive, **(B)** dominant, **(C)** over-dominant, and **(D)** additive models.

We analyzed the recessive model for rs211037, and the results indicated that the study was heterogeneous (*I* ^*2*^ = 73%). Because *I* ^*2*^ > 50% in the study, a random-effects model was chosen. The analysis was divided into Asian and non-Asian subgroups, and the results indicated that DRE was not linked to the Asian (OR = 0.99, 95% CI: 0.80–1.23, *p* = 0.94), non-Asian (OR = 0.19, 95% CI: 0.09–0.42, *p* < 0.01), and pooled populations (OR = 0.70, 95% CI: 0.40–1.22, *p* = 0.21) (Figure 7). According to subgroup analysis, the result indicated that the *GABRG2* rs211037 is not a genetic risk factor for DRE in Asian and non-Asian populations. We tested for publication bias according to subgroup analysis and found that there was no publication bias (Figure 8A; Table 4).

#### 3.4.3 Meta-analysis of the dominant model

**FIGURE 9:**
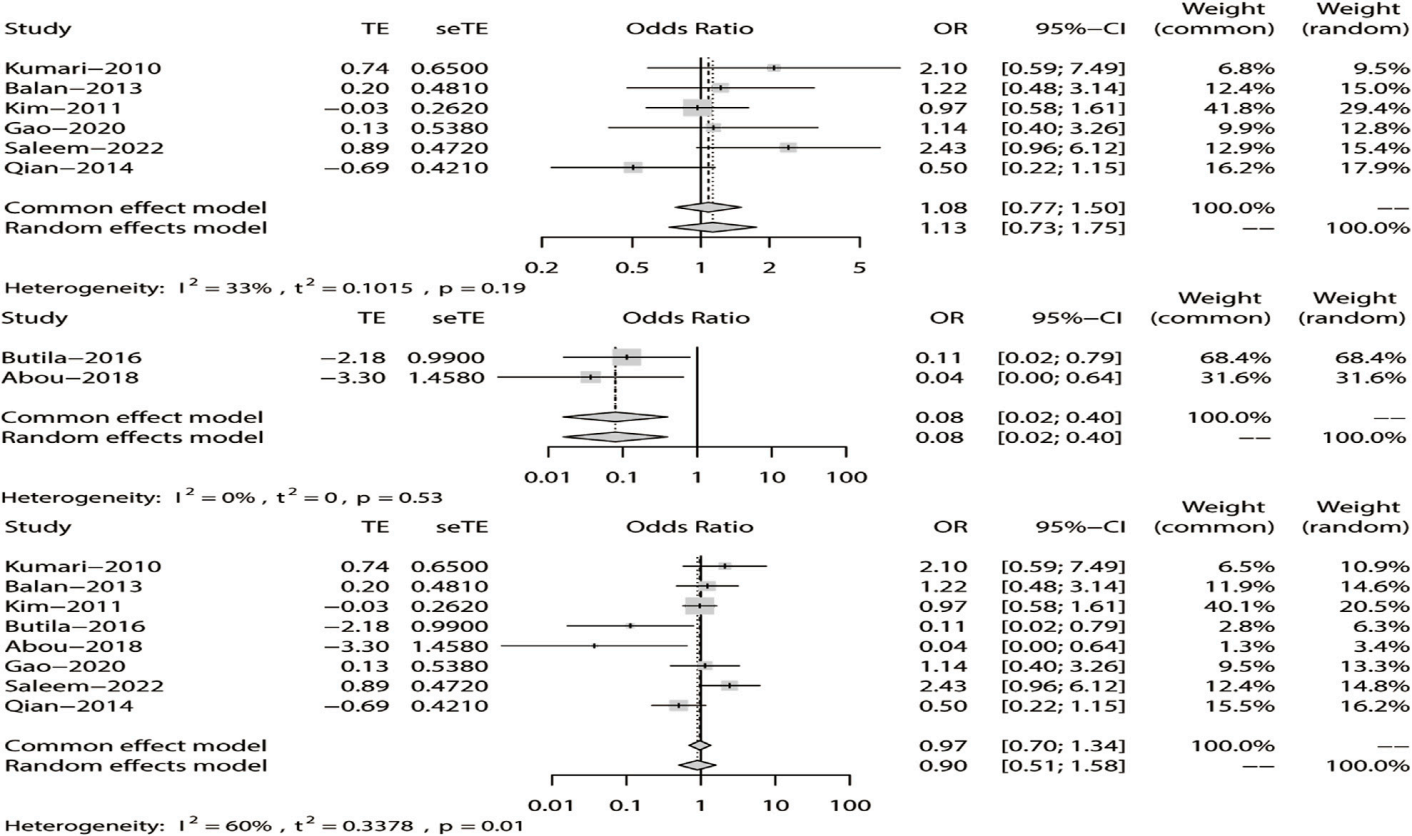
Random-effects meta-analysis of the dominant model for rs211037 in the Asian, non-Asian, and pooled populations.

Similarly, we used a random-effects model based on the dominant model of rs211037. This model indicated that rs211037 and DRE in the non-Asian (OR = 0.08, 95% CI: 0.02–0.40, *p* < 0.01) and pooled populations (OR = 0.90, 95% CI: 0.51–1.58, *p* = 0.71) were not closely related (Figure 9). Interestingly, however, an opposite result was found in the Asian population, where rs211037 was significantly correlated with DRE risk (OR = 1.08, 95% CI: 0.77–1.50, *p* = 0.65, Figure 9). Moreover, we did not detect any publication bias (Figure 8B; Table 4).

#### 3.4.4 Meta-analysis of the over-dominant model

**FIGURE 10:**
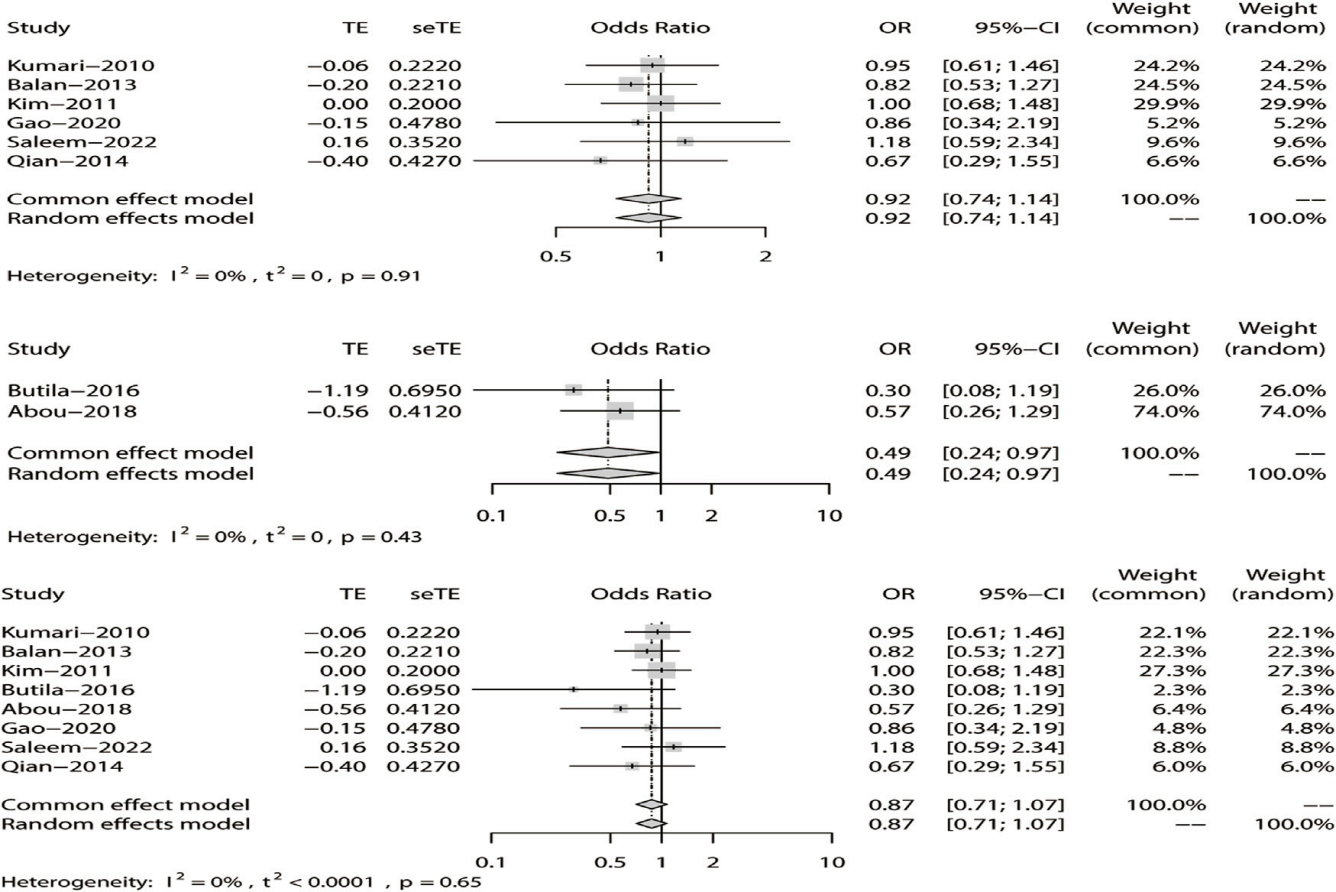
Fixed-effects meta-analysis of the over-dominant model for rs211037 in the Asian, non-Asian, and pooled populations.

Likewise, a fixed-effects model was selected to analyze this model. The tests suggested that DRE was unrelated to rs211037 in the Asian (OR = 0.92, 95% CI: 0.74–1.14, *p* = 0.46), non-Asian (OR = 0.49, 95% CI: 0.24–0.97, *p* = 0.04), and pooled populations (OR = 0.87, 95% CI: 0.71–1.07, *p* = 0.19) for this model (Figure 10). However, we tested for publication bias according to subgroup analysis and found that publication bias existed (Table 4; Figure 8C). Therefore, these results should be applied cautiously.

#### 3.4.5 Meta-analysis of the additive model

**FIGURE 11:**
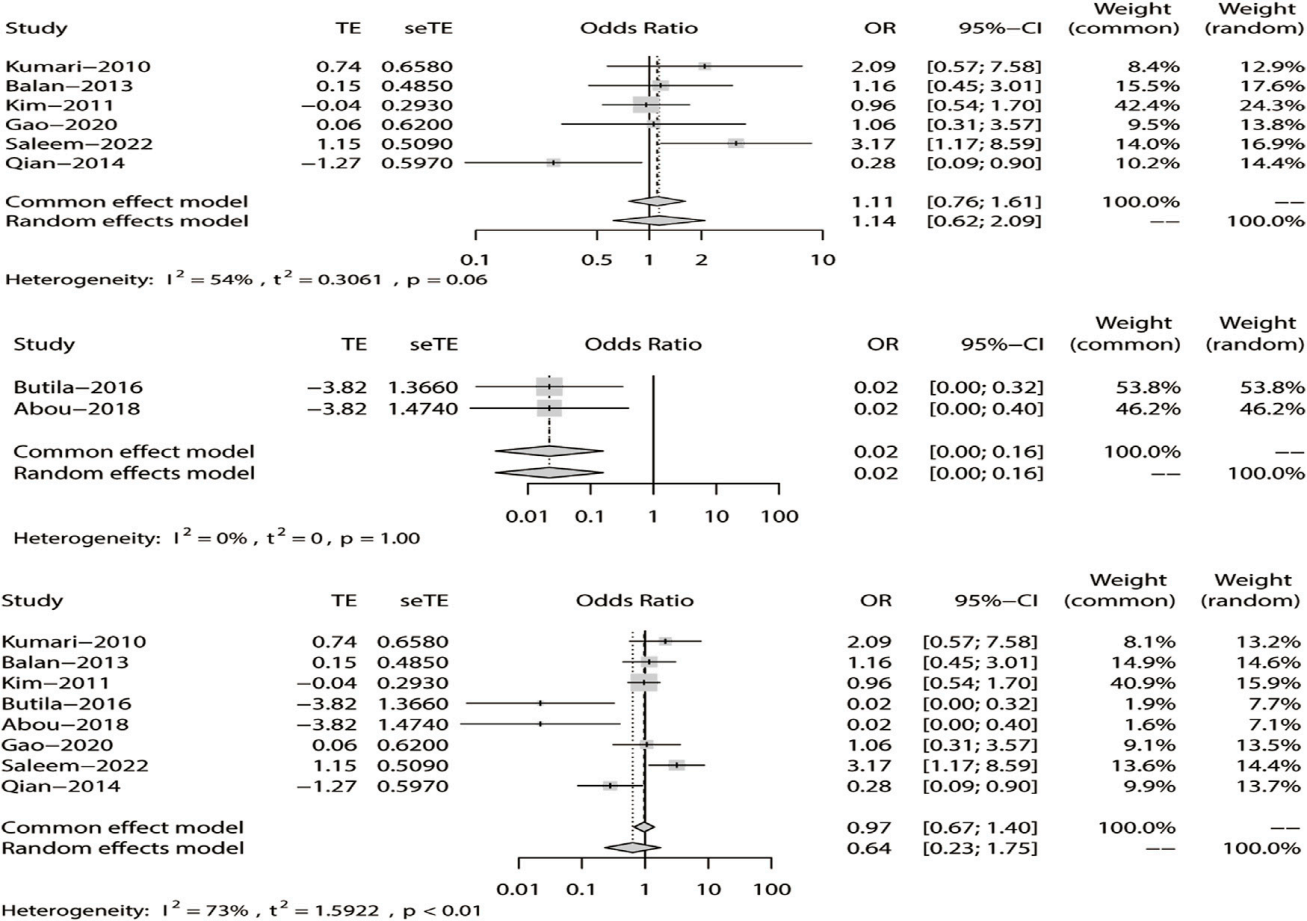
Random-effects meta-analysis of the additive model for rs211037 in the Asian, non-Asian, and pooled populations.

Finally, in the additive model, the random-effect model was chosen to analyze the overall OR (*I* ^*2*^ = 73%). The outcome indicated that there was no relationship between DRE and rs211037 in the non-Asian (OR = 0.02, 95% CI: 0.00–0.16, *p* < 0.01) and pooled populations (OR = 0.64, 95% CI: 0.23–1.75, *p* = 0.38) (Figure 11). However, rs211037 was significantly correlated with DRE risk in Asian populations (OR = 1.14, 95% CI: 0.62–2.09, *p* = 0.67) (Figure 11). We did not detect any publication bias (Table 4; Figure 8D).

## 4 Discussion

Epilepsy is caused by super-synchronized discharges of neurons in the brain, resulting in sudden and repeated short bursts of dysfunction of the central nervous system (Manford, 2017). With the increasing incidence of epilepsy, the treatment of epilepsy has gradually changed from a single drug to combinations of drugs, resulting in the occurrence of drug-resistant epilepsy in the clinical work. Hyperexcitability of neurons, due to an imbalance of inhibitory and excitatory neurotransmission, plays a crucial role in neuronal degeneration complicated by epilepsy (Shao et al., 2019). Important pharmacological targets that regulate neuronal activity in the brain are thought to be affected by mutations in ion channel genes (Saleem et al., 2022). Traditionally, epilepsy is treated by oral drugs and surgery, but with the development of genetic research, single-nucleotide polymorphism (SNP) markers can provide a new method to classify complex gene-related diseases, such as epilepsy and even drug-resistant epilepsy. This article mainly analyzes the relationship between transporters and drug-resistant epilepsy.

GABA is a major inhibitory neurotransmitter mainly found in the central nervous system of mammals, which can clear GABA from the synaptic cleft (Xie et al., 2017). GAT-3 (*SLC6A11*) is a GABA transport protein, and some studies have proved that epileptic activity leads to the change in expression of GAT-3 (Madsen et al., 2010). As GABAergic neurotransmission is terminated by uptake into the neuron or surrounding glial cells, inhibition of the GABA transporters responsible for uptake would prolong the GABAergic signal in a use-dependent manner, thereby counteracting GABAergic hypoactivity (Xie et al., 2017). Affecting the promoter activity or leading to the synthesis of protein products with the same amino acid sequence but different structural and functional properties may prevent the reversal of GAT-3 transporters, releasing GABA into the synaptic pool and resulting in decreased GABA energy, thereby protecting neurons from overexcitation, leading to AED resistance during seizures (Kim D. U. et al., 2011). Thus, inhibition of GABA transport has gained much attention as an anticonvulsive strategy (Xie et al., 2017). Most previous studies of resistance to AEDs have focused on ABC transporters or voltage-gated sodium channels. Little attention has been paid to the new candidate susceptibility gene *SLC6A11*. Thus, we can infer that GAT-3 is a potential target of DRE. This article assessed whether the *SLC6A11* rs2304725 polymorphism is associated with DRE. Herein, we showed that *SLC6A11* polymorphism was unlinked to DRE. In the analysis of rs2304725, we included four articles and concluded that rs2304725 had no significant correlation with drug-resistant epilepsy. This is consistent with the conclusion of the study by Kim D. U. et al. (2011). Researchers found that the expression of the GABA_A_ receptor subunit in drug-resistant rats was different from that in drug-responsive rats in a temporal lobe epilepsy rat model in 2008 (Bethmann et al., 2008). The result suggested that drug-resistant epilepsy may be largely related to GABA. Epilepsy, growth retardation, and behavioral disorders may be related to pathogenic *GABRG2* variants. GABRG2 variants may alter the expression of subunits of GABA_A_ receptors (the mechanism is unclear), which may affect transcription, mRNA stability, and translation efficiency, leading to variations in receptor composition and its sensitivity to exogenous environmental signals (Abou El Ella et al., 2018). rs211037 is a synonymous SNP of the γ-2 subunit of the GABA_A_ receptor (Amjad et al., 2022). Some studies have shown that the *GABRG2* gene may be correlated with both epilepsy and drug-resistant epilepsy and so we conducted some meta-analyses of *GABRG2* rs211037.

## 5 Limitation of the meta-analysis

First, in view of the research on rs2304725 and rs211037, some scholars have reported that rs2304725 and rs211037 are correlated with DRE, but there have also been contradictory conclusions; the possible reason is that the sample sizes included in the studies are small. Then, the selected research objects consist of mixed populations. Even if the Asian and non-Asian populations are analyzed, they are also possible sources of bias and mixing factors in the experiment, thus leading to the deviation of the experiment. Third, phenotypic heterogeneity and efficacy of antiepileptic drugs are also confounding factors in the meta-analysis. Lastly, the meta-analysis was conducted on the basis of other researchers’ studies, which can only reflect the historical situation and has low requirements for the accuracy and completeness of statistical data. In addition, the analysis was not carried out in combination with our own research, which is also a limitation to this paper.

## 6 Suggestions

This paper describes the mechanism of drug-resistant epilepsy induced by GAT-3 and GABA genes. However, a more detailed explanation of the potential mechanisms of DRE caused by the rs2304725 and rs211037 SNPs cannot be provided. Animal models can be developed to explore the underlying mechanisms. They may be more closely related to specific types of epilepsy, so different types of epilepsy can be studied in the future. We should also include large samples and populations for further study and analysis to clarify whether rs2304725 and rs211037 are linked to DRE. At the same time, the population difference was further analyzed. Finally, we should combine our own research to make the analysis more authentic and reliable.

## 7 Conclusion

Our results indicate that *SLC6A11* rs2304725 and *GABRG2* rs211037 are not associated with DRE for the allele model. rs2304725 was also not correlated with DRE for the dominant, recessive, and additive models. However, in the over-dominant model, rs2304725 was significantly correlated with DRE. Likewise, *GABRG2* rs211037 conveyed genetic risk for DRE in the Asian population in the allele, dominant, and additive models, whereas rs211037 had no significant correlation with DRE in the other models.

## Data Availability

The datasets presented in this study can be found in online repositories. The names of the repository/repositories and accession number(s) can be found in the article/Supplementary Material.

## Supplementary material

SUPPLEMENTARY TABLE S1 Search strategy for rs2304725 and rs211037 in this meta-analysis.

## References

[B1] Abou El Ella S. S. Tawfik M. A. Abo El Fotoh W. M. M. Soliman O. A. M. (2018). The genetic variant "C588T" of GABARG2 is linked to childhood idiopathic generalized epilepsy and resistance to antiepileptic drugs. Seizure 60, 39–43. 10.1016/j.seizure.2018.06.004 29894917

[B2] Amjad M. Tabassum A. Sher K. Kumar S. Zehra S. Fatima S. (2022). Impact of GABA(A) receptor gene variants (rs2279020 and rs211037) on the risk of predisposition to epilepsy: A case-control study. Neurological Sci. official J. Italian Neurological Soc. Italian Soc. Clin. Neurophysiology 43 (7), 4431–4438. 10.1007/s10072-022-05947-7 35150350

[B3] Balan S. Sathyan S. Radha S. K. Joseph V. Radhakrishnan K. Banerjee M. (2013). GABRG2, rs211037 is associated with epilepsy susceptibility, but not with antiepileptic drug resistance and febrile seizures. Pharmacogenetics genomics 23 (11), 605–610. 10.1097/fpc.0000000000000000 24061200

[B4] Bethmann K. Fritschy J. M. Brandt C. Löscher W. (2008). Antiepileptic drug resistant rats differ from drug responsive rats in GABA A receptor subunit expression in a model of temporal lobe epilepsy. Neurobiol. Dis. 31 (2), 169–187. 10.1016/j.nbd.2008.01.005 18562204

[B5] Butilă A. T. Zazgyva A. Sin A. I. Szabo E. R. Tilinca M. C. (2018). GABRG2 C588T gene polymorphisms might be a predictive genetic marker of febrile seizures and generalized recurrent seizures: A case-control study in a Romanian pediatric population. Archives Med. Sci. AMS 14 (1), 157–166. 10.5114/aoms.2016.63739 PMC5778423 29379546

[B6] Fisher R. S. van Emde Boas W. Blume W. Elger C. Genton P. Lee P. (2005). Epileptic seizures and epilepsy: Definitions proposed by the international League against epilepsy (ILAE) and the international bureau for epilepsy (IBE). Epilepsia 46 (4), 470–472. 10.1111/j.0013-9580.2005.66104.x 15816939

[B7] Galvan A. Villalba R. M. West S. M. Maidment N. T. Ackerson L. C. Smith Y. (2005). GABAergic modulation of the activity of globus pallidus neurons in primates: *In vivo* analysis of the functions of GABA receptors and GABA transporters. J. neurophysiology 94 (2), 990–1000. 10.1152/jn.00068.2005 15829599

[B8] Gao H. Qi H. Mao J. Yang X. (2020). Association between the polymorphism of GABA(A) receptor gene and epilepsy in Uygur in Xinjiang. J. Shanxi Med. Univ. 51 (07), 711–715. 10.13753/j.issn.1007-6611.2020.07.018

[B9] Haerian B. S. Baum L. (2013). GABRG2 rs211037 polymorphism and epilepsy: A systematic review and meta-analysis. Seizure 22 (1), 53–58. 10.1016/j.seizure.2012.10.007 23140995

[B10] Higgins Jpt T. J. Thomas C. J. Cumpston M. Li T. Page M. J. Welch V. A. (2021). Cochran handbook for systematic reviews of interventions version 6.2.

[B11] Hu Y. Zheng L. Cheng L. Zhang Y. Bai W. Zhou W. (2017). GAB2 rs2373115 variant contributes to Alzheimer's disease risk specifically in European population. J. neurological Sci. 375, 18–22. 10.1016/j.jns.2017.01.030 28320126

[B12] Kalilani L. Sun X. Pelgrims B. Noack-Rink M. Villanueva V. (2018). The epidemiology of drug-resistant epilepsy: A systematic review and meta-analysis. Epilepsia 59 (12), 2179–2193. 10.1111/epi.14596 30426482

[B13] Kim D. U. Kim M. K. Cho Y. W. Kim Y. S. Kim W. J. Lee M. G. (2011a). Association of a synonymous GAT3 polymorphism with antiepileptic drug pharmacoresistance. J. Hum. Genet. 56 (9), 640–646. 10.1038/jhg.2011.73 21776001

[B14] Kim M. K. Moore J. H. Kim J. K. Cho K. H. Cho Y. W. Kim Y. S. (2011b). Evidence for epistatic interactions in antiepileptic drug resistance. J. Hum. Genet. 56 (1), 71–76. 10.1038/jhg.2010.151 21124337

[B15] Kumari R. Lakhan R. Kalita J. Misra U. K. Mittal B. (2010). Association of alpha subunit of GABAA receptor subtype gene polymorphisms with epilepsy susceptibility and drug resistance in north Indian population. Seizure 19 (4), 237–241. 10.1016/j.seizure.2010.02.009 20356767

[B16] Kwan P. Arzimanoglou A. Berg A. T. Brodie M. J. Allen Hauser W. Mathern G. (2010). Definition of drug resistant epilepsy: Consensus proposal by the ad hoc task force of the ILAE commission on therapeutic strategies. Epilepsia 51 (6), 1069–1077. 10.1111/j.1528-1167.2009.02397.x 19889013

[B17] Liu G. Zhang S. Cai Z. Ma G. Zhang L. Jiang Y. (2013). PICALM gene rs3851179 polymorphism contributes to Alzheimer's disease in an Asian population. Neuromolecular Med. 15 (2), 384–388. 10.1007/s12017-013-8225-2 23572399

[B18] Liu G. Wang H. Liu J. Li J. Li H. Ma G. (2014). The CLU gene rs11136000 variant is significantly associated with Alzheimer's disease in Caucasian and Asian populations. Neuromolecular Med. 16 (1), 52–60. 10.1007/s12017-013-8250-1 23892938

[B19] Liu G. Xu Y. Jiang Y. Zhang L. Feng R. Jiang Q. (2017). PICALM rs3851179 variant confers susceptibility to alzheimer's disease in Chinese population. Mol. Neurobiol. 54 (5), 3131–3136. 10.1007/s12035-016-9886-2 27048444

[B20] Long H. (2014). Association study between the polymorphisms of SLC6A11 and APOE gene and drug-resistant epilepsy in Chinese Han population. Central south university.

[B21] Löscher W. Potschka H. Sisodiya S. M. Vezzani A. (2020). Drug resistance in epilepsy: Clinical impact, potential mechanisms, and new innovative treatment options. Pharmacol. Rev. 72 (3), 606–638. 10.1124/pr.120.019539 32540959 PMC7300324

[B22] Macdonald R. L. Gallagher M. J. Feng H. J. Kang J. (2004). GABA(A) receptor epilepsy mutations. Biochem. Pharmacol. 68 (8), 1497–1506. 10.1016/j.bcp.2004.07.029 15451392

[B23] Madsen K. K. White H. S. Schousboe A. (2010). Neuronal and non-neuronal GABA transporters as targets for antiepileptic drugs. Pharmacol. Ther. 125 (3), 394–401. 10.1016/j.pharmthera.2009.11.007 20026354

[B24] Maljevic S. Møller R. S. Reid C. A. Pérez-Palma E. Lal D. May P. (2019). Spectrum of GABAA receptor variants in epilepsy. Curr. Opin. neurology 32 (2), 183–190. 10.1097/wco.0000000000000657 30664068

[B25] Manford M. (2017). Recent advances in epilepsy. J. neurology 264 (8), 1811–1824. 10.1007/s00415-017-8394-2 PMC5533817 28120042

[B26] Meldrum B. S. Rogawski M. A. (2007). Molecular targets for antiepileptic drug development. Neurother. J. Am. Soc. Exp. Neurother. 4 (1), 18–61. 10.1016/j.nurt.2006.11.010 PMC1852436 17199015

[B27] Mizielinska S. Greenwood S. Connolly C. N. (2006). The role of GABAA receptor biogenesis, structure and function in epilepsy. Biochem. Soc. Trans. 34 (5), 863–867. 10.1042/bst0340863 17052216

[B28] Moshé S. L. (2000). Mechanisms of action of anticonvulsant agents. Neurology 55 (5), S32–S40. discussion S54-8. 11001360

[B29] Ms H. (2016). The impact of genetic polymorphisms in candidate genes on susceptibility to epilepsy and responsiveness to antiepileptics in patients with epilepsy/Hidayati Mohd Sha'ari. Kuala Lumpur, Malaysia: University of Malaya.

[B30] Mulligan M. K. Wang X. Adler A. L. Mozhui K. Lu L. Williams R. W. (2012). Complex control of GABA(A) receptor subunit mRNA expression: Variation, covariation, and genetic regulation. PloS one 7 (4), e34586. 10.1371/journal.pone.0034586 22506031 PMC3323555

[B31] Oyrer J. Maljevic S. Scheffer I. E. Berkovic S. F. Petrou S. Reid C. A. (2018). Ion channels in genetic epilepsy: From genes and mechanisms to disease-targeted therapies. Pharmacol. Rev. 70 (1), 142–173. 10.1124/pr.117.014456 29263209 PMC5738717

[B32] Qian Z. (2017). Study on the relationship between single nucleotide polymorphism of GABAA receptor subunit gene and refractory epilepsy(in Chinese). Youjiang Med. Coll. Natl. 80.

[B33] Saleem T. Maqbool H. Sheikh N. Tayyeb A. Mukhtar M. Ashfaq A. (2022). GABRG2 C588T polymorphism is associated with idiopathic generalized epilepsy but not with antiepileptic drug resistance in Pakistani cohort. BioMed Res. Int. 2022, 3460792. 10.1155/2022/3460792 36425336 PMC9681559

[B34] Shao L. R. Habela C. W. Stafstrom C. E. (2019). Pediatric epilepsy mechanisms: Expanding the paradigm of excitation/inhibition imbalance. Child. (Basel, Switz. 6 (2), 23. 10.3390/children6020023 PMC6406372 30764523

[B35] Shen D. Hernandez C. C. Shen W. Hu N. Poduri A. Shiedley B. (2017). De novo GABRG2 mutations associated with epileptic encephalopathies. Brain a J. neurology 140 (1), 49–67. 10.1093/brain/aww272 PMC5226060 27864268

[B36] Sisodiya S. M. (2005). Genetics of drug resistance in epilepsy. Curr. neurology Neurosci. Rep. 5 (4), 307–311. 10.1007/s11910-005-0076-2 15987615

[B37] Staley K. (2015). Molecular mechanisms of epilepsy. Nat. Neurosci. 18 (3), 367–372. 10.1038/nn.3947 25710839 PMC4409128

[B38] Thijs R. D. Surges R. O'Brien T. J. Sander J. W. (2019). Epilepsy in adults. Lancet (London, Engl. 393 (10172), 689–701. 10.1016/s0140-6736(18)32596-0 30686584

[B39] Wang Y. Zhang S. Li F. Zhou Y. Zhang Y. Wang Z. (2020). Therapeutic target database 2020: Enriched resource for facilitating research and early development of targeted therapeutics. Nucleic acids Res. 48 (D1), D1031–d1041. 10.1093/nar/gkz981 31691823 PMC7145558

[B40] Wang D. Hu X. Yin X. Cui C. Yang X. Li Y. (2022). Effectiveness of thalidomide for ankylosing spondylitis: A meta-analysis of randomized controlled trials in China. Clin. Rheumatol. 41 (10), 2929–2938. 10.1007/s10067-022-06220-0 35635651

[B41] Wishart D. S. Feunang Y. D. Guo A. C. Lo E. J. Marcu A. Grant J. R. (2018). DrugBank 5.0: A major update to the DrugBank database for 2018. Nucleic acids Res. 46 (D1), D1074–d1082. 10.1093/nar/gkx1037 29126136 PMC5753335

[B42] Xie Y. Y. Qu J. Zhou L. Lv N. Gong J. E. Cao Y. Z. (2017). Lack of association between SLC6A11 genetic polymorphisms and drug resistant epilepsy in Chinese han population. Clin. Lab. 63 (7), 1113–1120. 10.7754/Clin.Lab.2017.161217 28792706

[B43] Yu Sun L. L. Li L. Wang J. (2021). An advance about the genetic causes of epilepsy. E3S Web Conf. 271, 8. 10.1051/e3sconf/202127103068

[B44] Zhang X. Liu J. Ye J. (2021). Association between SCN1A polymorphism and carbamazepine responsiveness in epilepsy: A meta-analysis. Epilepsy Res. 176, 106627. 10.1016/j.eplepsyres.2021.106627 34218210

