# Supplementary material for: Association between the SLC6A11 rs2304725 and GABRG2 rs211037 polymorphisms and drug-resistant epilepsy: a meta-analysis
Source: Front Physiol. 2023 May 19;14:1191927. doi: 10.3389/fphys.2023.1191927 (PMC10235491; doi:10.3389/fphys.2023.1191927)
Supplement: Supplementary file 1 [file Table1.docx]

**Supplementary Tables**

**Table 1** Search strategies

| **Step** | **Searching Strategy** | **No. of articles** |
| --- | --- | --- |
| ***PubMed*** | | |
| #1 | Search "Drug Resistant Epilepsy"[Mesh] | 3461 |
| #2 | Search Drug Resistant Epilepsies OR Epilepsies, Drug Resistant OR Resistant Epilepsies, Drug OR Resistant Epilepsy, Drug OR Epilepsy, Drug Resistant OR Medication Resistant Epilepsy OR Epilepsies, Medication Resistant OR Epilepsy, Medication Resistant OR Medication Resistant Epilepsies OR Resistant Epilepsies, Medication OR Resistant Epilepsy, Medication OR Intractable Epilepsy OR Epilepsies, Intractable OR Intractable Epilepsies OR Epilepsy, Drug Refractory OR Epilepsy, Intractable OR Refractory Epilepsy OR Epilepsies, Refractory OR Epilepsy, Refractory OR Refractory Epilepsies OR Drug Refractory Epilepsy OR Drug Refractory Epilepsies OR Epilepsies, Drug Refractory OR Refractory Epilepsies, Drug OR Refractory Epilepsy, Drug | 23156 |
| #3 | Search #1 OR #2 | 23124 |
| #4 | Search "gaba transporter 3" | 1713 |
| #5 | Search (GAT-3) OR (rs2304725) OR (SLC6A11) | 271 |
| #6 | Search #4 OR #5 | 1791 |
| #7 | Search #3 AND #6 | 11 |
| #8 | Search "GABRG2"[Mesh] | 408 |
| #9 | Search (C588T) OR (rs211037) | 23 |
| #10 | Search #8 OR #9 | 414 |
| #11 | Search #3 AND #10 | 19 |
| ***Embase*** | | |
| #1 | Search 'drug resistant epilepsy'/exp | 7171 |
| #2 | Search (drug refractory epilepsy) OR (medication resistant epilepsy) OR (refractory epilepsy) | 18637 |
| #3 | Search #1 OR #2 | 22255 |
| #4 | Search 'gaba transporter 3'/exp | 275 |
| #5 | Search rs2304725 OR slc6a11 | 48 |
| #6 | Search GAT-3 | 259 |
| #7 | Search #4 OR #5 OR #6 | 480 |
| #8 | Search #3 AND #7 | 5 |
| #9 | Search ' gabrg2'/exp | 423 |
| #10 | Search c588t OR rs211037 | 30 |
| #11 | Search #9 OR #10 | 431 |
| #12 | Search #3 AND #11 | 13 |
| ***Cochrane Library*** | | |
| #1 | Search Mesh descriptor: [Drug Resistance Epilepsy] ti,ab,kw | 825 |
| #2 | Search ('Drug Resistant Epilepsies')ti,ab,kw OR ('Epilepsies, Drug Resistant'):ti,ab,kw OR ('Resistant Epilepsies, Drug'):ti,ab,kw OR ('Resistant Epilepsy, Drug'):ti,ab,kw OR ('Epilepsy, Drug Resistant'):ti,ab,kw OR ('Medication Resistant Epilepsy'):ti,ab,kw OR ('Epilepsies, Medication Resistant'):ti,ab,kw OR ('Epilepsy, Medication Resistant'):ti,ab,kw OR ('Medication Resistant Epilepsies'):ti,ab,kw OR ('Resistant Epilepsies, Medication'):ti,ab,kw OR ('Resistant Epilepsy, Medication'):ti,ab,kw OR ('Intractable Epilepsy'):ti,ab,kw OR ('Epilepsies, Intractable'):ti,ab,kw OR ('Intractable Epilepsies'):ti,ab,kw OR ('Epilepsy, Drug Refractory'):ti,ab,kw OR ('Epilepsy, Intractable'):ti,ab,kw OR ('Refractory Epilepsy'):ti,ab,kw OR ('Epilepsy, Refractory'):ti,ab,kw OR (' Epilepsies, Refractory'):ti,ab,kw OR ('Refractory Epilepsies'):ti,ab,kw OR ('Drug Refractory Epilepsy'):ti,ab,kw OR ('Drug Refractory Epilepsies'):ti,ab,kw OR ('Epilepsies, Drug Refractory'):ti,ab,kw OR ('Refractory Epilepsies, Drug'):ti,ab,kw OR ('Refractory Epilepsy, Drug '):ti,ab,kw | 1840 |
| #3 | Search #1 OR #2 | 1899 |
| #4 | Search gaba transporter 3 | 37 |
| #5 | Search (rs2304725) OR (gat-3) OR (SLC6A11) | 4 |
| #6 | Search #4 OR #5 | 41 |
| #7 | Search #3 AND #6 | 1 |
| #8 | Search gabrg2 | 2 |
| #9 | Search (C588T) OR (rs211370) | 0 |
| #10 | Search #8 OR #9 | 2 |
| #8 | Search #3 AND #10 | 0 |
| ***Web of Science*** | | |
| #1 | Search theme: (drug resistance epilepsy） | 1687 |
| #2 | Search theme: (drug refractory epilepsy) OR theme: (medication resistant epilepsy) OR theme: (refractory epilepsy) | 11927 |
| #3 | Search #1 OR #2 | 13087 |
| #4 | Search theme: (gaba transporter 3) | 1473 |
| #5 | Search theme: (GAT-3) OR theme: (rs2304725) OR theme: (SLC6A11） | 223 |
| #6 | Search #4 OR #5 | 1525 |
| #7 | Search #3 AND #6 | 14 |
| #8 | Search theme:(GABRG2) | 267 |
| #9 | Search theme:(C588T) OR theme:(rs211370) | 8 |
| #10 | Search #8 OR #9 | 268 |
| #11 | Search #3 AND #10 | 13 |
|  | ***Google Scholar*** |  |
| #1 | Search: (GAT-3 OR rs2304725 OR SLC6A11) AND (drug resistance epilepsy) | 45 |
| #2 | Limitations: Clinical studies | 13 |
| #3 | Search : (GABRG2 or rs211037 or C588T) AND (drug resistance epilepsy) | 136 |
| #4 | Limitations: Clinical studies | 93 |
|  | ***China National Knowledge Infrastructure (CNKI)*** |  |
| #1 | Search theme: SLC6A11+GAT-3+rs2304725 AND theme: drug resistance epilepsy (in Chinese) | 3 |
| #2 | Limitations: Clinical studies（in Chinese） | 2 |
| #3 | Search theme: GABRG2+rs211037+C588T AND theme: drug resistance epilepsy (in Chinese) | 5 |
| #4 | Limitations: Clinical studies（in Chinese） | 1 |
|  | ***Wanfang Data*** |  |
| #1 | Search theme: drug resistance epilepsy AND theme：rs211037 or C588T or GABGR2（in Chinese） | 1 |
| #2 | Limitations: Clinical studies（in Chinese） | 1 |
| #3 | Search theme: drug resistance epilepsy AND theme：GAT-3 or rs2304725 or SLC6A11（in Chinese） | 4 |
| #4 | Limitations: Clinical studies（in Chinese） | 2 |
|  | ***VIP*** |  |
| #1 | Search keyword: drug resistance epilepsy AND keyword：GAT-3 or rs2304725 or SLC6A11（in Chinese） | 1 |
| #2 | Limitations: Clinical studies（in Chinese） | 1 |
| #3 | Search keyword: drug resistance epilepsy AND keyword：rs211037 or C588T or GABGR2（in Chinese） | 2 |
| #4 | Limitations: Clinical studies（in Chinese） | 2 |
